# OA26 Anti-NXP2-Positive Juvenile Dermatomyositis in an Adolescent Following a Five-Year History of Childhood Cutaneous Manifestations, Presenting with Severe Myositis, Bulbar Weakness and Respiratory Failure Complicated by Macrophage Activation Syndrome

**DOI:** 10.1093/rap/rkag113.026

**Published:** 2026-11-03

**Authors:** Md Shahriar Kabir, Maira Karabayas, Paula Dospinescu

**Affiliations:** NHS Grampian Rheumatology Department, Aberdeen, United Kingdom; NHS Grampian Rheumatology Department, Aberdeen, United Kingdom; NHS Grampian Rheumatology Department, Aberdeen, United Kingdom

## Abstract

**Introduction:**

Juvenile dermatomyositis (JDM) is a rare autoimmune inflammatory myopathy of childhood characterised by proximal muscle weakness and characteristic cutaneous manifestations. Cutaneous features may precede muscle involvement by months or even years, posing a diagnostic challenge. Anti-NXP2-positive JDM is associated with more severe muscle disease and an increased risk of systemic complications. We present a teenager with a five-year history of intermittent photosensitive facial rash who subsequently developed rapidly progressive JDM complicated by macrophage activation syndrome, severe bulbar dysfunction and respiratory failure requiring intensive care, with an excellent clinical recovery following timely escalation of immunosuppressive therapy and multidisciplinary management

**Case description:**

We report the case of a 17-year-old boy with a five-year history of an intermittent photosensitive facial rash with seasonal exacerbations. Dermatological assessment demonstrated periorbital and periungual erythema without classical Gottron papules or a shawl sign. Skin biopsy initially raised the possibility of cutaneous lupus but remained non-specific, highlighting the importance of clinicopathological correlation. At 17 years of age, he developed rapidly progressive proximal muscle weakness, myalgia and dysphagia over eight weeks, necessitating hospital admission.

Given the rapidly progressive weakness, evolving bulbar involvement and creatine kinase (CK) >31,000 U/L, juvenile dermatomyositis was suspected. Intravenous methylprednisolone was commenced promptly, and following discussion with the regional paediatric rheumatology team, intravenous immunoglobulin (IVIG) was initiated while diagnostic investigations were ongoing. MRI demonstrated diffuse symmetrical muscle oedema, muscle biopsy showed necrotising myopathy, and myositis immunoblot confirmed anti-NXP2 antibody positivity, establishing the diagnosis of juvenile dermatomyositis.

Following an initial clinical and biochemical response, weekly subcutaneous methotrexate and rituximab were introduced. However, he subsequently deteriorated with progressive bulbar dysfunction requiring nasogastric feeding and worsening proximal muscle weakness, progressing to respiratory failure requiring intensive care admission and invasive mechanical ventilation. His admission was further complicated by right lower lobe pneumonia, *Klebsiella* urinary tract infection and failed extubation secondary to severe bulbar weakness. During his intensive care admission, he developed persistent sinus tachycardia with progressive thrombocytopenia, hyperferritinaemia, hypertriglyceridaemia and elevated lactate dehydrogenase, consistent with macrophage activation syndrome (MAS). Timely escalation of immunosuppression with further intravenous methylprednisolone, additional IVIG and ciclosporin, alongside coordinated multidisciplinary management, resulted in sustained clinical and biochemical recovery. Following successful extubation, he underwent inpatient rehabilitation with physiotherapy, speech and language therapy, occupational therapy and dietetic input, ultimately returning to his pre-morbid level of function with restoration of swallowing and muscle strength.

**Discussion:**

Recognition of juvenile dermatomyositis can be challenging when cutaneous manifestations precede muscle involvement by several years. This case illustrates how a prolonged atypical cutaneous prodrome posed a diagnostic challenge before progressing to severe anti-NXP2-positive juvenile dermatomyositis complicated by macrophage activation syndrome (MAS).

Once inflammatory myopathy became clinically apparent, the disease progressed rapidly despite an initial response to treatment, culminating in severe bulbar dysfunction, respiratory failure and MAS. This reflects the aggressive phenotype recognised in anti-NXP2-positive juvenile dermatomyositis and reinforces the importance of early specialist involvement and initiating immunosuppressive therapy based on clinical suspicion, rather than waiting for complete diagnostic confirmation.

MAS remains an uncommon but potentially fatal complication of juvenile dermatomyositis. In our patient, evolving thrombocytopenia, hyperferritinaemia and hypertriglyceridaemia prompted early recognition of MAS and urgent escalation of immunosuppression. Despite prolonged intensive care support, coordinated management between paediatric rheumatology, intensive care and the wider multidisciplinary team resulted in sustained neurological recovery and an excellent functional outcome.

Ultimately, this case serves as a reminder that persistent atypical photosensitive rashes in children should prompt continued surveillance for evolving inflammatory myopathy, even when initial investigations are inconclusive. Early recognition of aggressive disease, prompt escalation of immunosuppressive therapy and coordinated multidisciplinary care can achieve excellent outcomes, even in patients with life-threatening juvenile dermatomyositis requiring intensive care support

**Key learning points:**

1. Atypical cutaneous juvenile dermatomyositis may precede muscle involvement by several years, highlighting the importance of clinicopathological correlation and continued clinical surveillance in children with persistent photosensitive rashes.

2. In rapidly progressive juvenile dermatomyositis with evolving bulbar involvement, treatment should not be delayed while awaiting diagnostic confirmation. Early specialist paediatric rheumatology input and prompt initiation of immunosuppressive therapy may be crucial.

3. Anti-NXP2-positive juvenile dermatomyositis may follow an aggressive disease course with severe muscle involvement and life-threatening complications, including macrophage activation syndrome (MAS) and respiratory failure. Early recognition of MAS and timely escalation of immunosuppressive therapy are essential to improving outcomes.

4. Coordinated multidisciplinary management, including regional paediatric rheumatology expertise, intensive care, physiotherapy, speech and language therapy, occupational therapy and dietetic support, is fundamental to achieving excellent functional recovery despite critical illness.

